# How Similar Are Drug Data and Disease Self-report? Estimating the Prevalence of Chronic Diseases in Less Developed Settings

**DOI:** 10.34172/aim.27553

**Published:** 2024-07-01

**Authors:** Seyed Reza Abdipour Mehrian, Zahra Ghahramani, Mohammad Reza Akbari, Elham Hashemi, Ehsan Shojaeefard, Reza Malekzadeh, Bita Mesgarpour, Abdullah Gandomkar, Mohammad Reza Panjehshahin, Jafar Hasanzadeh, Fatemeh Malekzadeh, Hossein Molavi Vardanjani

**Affiliations:** ^1^MD-MPH Program, School of Medicine, Shiraz University of Medical Sciences, Shiraz, Iran; ^2^Hematology Research Center, Shiraz University of Medical Sciences, Shiraz, Iran; ^3^Liver, Pancreatic, and Biliary Diseases Research Center, Digestive Diseases Research Institute, Tehran University of Medical Sciences, Tehran, Iran; ^4^Vice Chancellery for Research & Technology, Iran Ministry of Health and Medical Education, Tehran, Iran; ^5^Non-Communicable Disease Research Center, Shiraz University of Medical Sciences, Shiraz, Iran; ^6^Faculty of Pharmacy, Shiraz University of Medical Science, Medicinal & Natural Products Chemistry Research Center, School of Medicine, Shiraz University of Medical Sciences, Shiraz, Iran; ^7^Department of Epidemiology, Shiraz University of Medical Sciences, Shiraz, Iran; ^8^Digestive Diseases Research Center, Digestive Diseases Research Institute, Shariati Hospital, Tehran University of Medical Sciences, Tehran, Iran; ^9^MD-MPH Program, School of Medicine, Research Center for Traditional Medicine and History of Medicine, Shiraz University of Medical Sciences, Shiraz, Iran

**Keywords:** Data source, Drug data, Prevalence, Self-report, Validity

## Abstract

**Background:**

Drug data has been used to estimate the prevalence of chronic diseases. Disease registries and annual surveys are lacking, especially in less-developed regions. At the same time, insurance drug data and self-reports of medications are easily accessible and inexpensive. We aim to investigate the similarity of prevalence estimation between self-report data of some chronic diseases and drug data in a less developed setting in southwestern Iran.

**Methods:**

Baseline data from the Pars Cohort Study (PCS) was re-analyzed. The use of disease-related drugs were compared against self-report of each disease (hypertension [HTN], diabetes mellitus [DM], heart disease, stroke, chronic obstructive pulmonary disease [COPD], sleep disorder, anxiety, depression, gastroesophageal reflux disease [GERD], irritable bowel syndrome [IBS], and functional constipation [FC]). We used sensitivity, specificity, positive predictive value (PPV), negative predictive value (NPV), and the Jaccard similarity index.

**Results:**

The top five similarities were observed in DM (54%), HTN (53%), heart disease (32%), COPD (30%), and GERD (15%). The similarity between drug use and self-report was found to be low in IBS (2%), stroke (5%), depression (9%), sleep disorders (10%), and anxiety disorders (11%).

**Conclusion:**

Self-reports of diseases and the drug data show a different picture of most diseases’ prevalence in our setting. It seems that drug data alone cannot estimate the prevalence of diseases in settings similar to ours. We recommend using drug data in combination with self-report data for epidemiological investigation in the less-developed setting.

## Introduction

 Different types of data have been used to estimate the prevalence of diseases. Self-report data, clinical examination, and paraclinical data are commonly used to find the prevalence of diseases. Like any other subjective data, self-report data is associated with inaccurate reporting due to recall bias, social desirability bias,^1^ and cognitive deficit. Low cost and practicality are advantages of this method.^2^ Clinical examination and paraclinical data are used for detection of diseases. These types of data are costly but more accurate. Although self-reporting serves as a crucial source of information for physicians in clinical settings, it is frequently observed that patients’ medical history does not align with their medication history. This challenges physicians to assess the reliability of their patients’ claims about having certain diseases. This clinical problem can have broader implications in epidemiology, particularly in studies that rely on self-reported disease data, such as prevalence surveys.

 Drug data has also been used to estimate the prevalence of diseases.^3,4^ It is objective, accessible, inexpensive, and majorly registered in insurance databases or health records. Health surveillance systems, disease registries, insurance databases, and regular national health surveys are available in more developed regions, while in less-developed areas, sustainable and integrated surveillance is lacking.^5,6^ Thus, we hypothesized that drug data is suitable in less-developed settings due to its feasibility and inexpensiveness.

 Some studies compared self-reports with medical records in the literature, and the results were contradictory.^7-10^ Only a few studies have compared medication data with other sources. A study by Chini et al showed that drug data could be used to provide reliable prevalence estimates of several chronic diseases. This study was conducted in Lazio, a region in central Italy. This study used data from one registry for drug data, which collects drug data of prescribed medications by general physicians or outpatient centers (not hospitals). Disease data were gathered from two registries that collect health records provided by health care units and a population-based survey that gathered self-reports of diseases.^3^ In another study, Hafferty et al compared the validity of self-reported certain drug use to national prescription data. They found that self-reported drugs were accurate compared to prescription data. This study used self-reported drug data from a cohort of Scottish adults. The prescribed drug data came from health records in the National Health Information Registry.^11^ However, we did not find a study that compared medication data with self-reported data.

 This study investigates the similarity of medication use to patients’ self-report of diseases. Here, we try to answer whether the drug data estimates the disease prevalence similar to self-report data. We investigated the similarity between self-reporting of chronic diseases and drug data, using sensitivity, specificity, positive predictive value (PPV), negative predictive value (NPV), and Jaccard similarity index.

## Materials and Methods

###  Study Design

 This is a cross-sectional study, and the data was obtained from the Pars Cohort Study (PCS). PCS started in 2014 and is still ongoing in Valashahr, the Fars province.

###  Setting

 Valashahr is a county of the Fars province in southern Iran. This area is a semi-urban area with 40 000 inhabitants. The inhabitants of this region are mostly of Turkish and Persian ethnicities. Primary health centers and general practitioners in the private sector provide health services in this area. Specialized and sub-specialized services are available in Fars’ surrounding cities and capital (Shiraz). More information about PCS can be found elsewhere.^12,13^

###  Data Collection

####  Demographic Data

 Using a standardized questionnaire, the PCS study gathered sex, age, ethnicity, education, and socioeconomic status (SES).^12^ Age is categorized into three groups: under 50, between 50 and 60, and over 60 years; ethnicity was tagged as Persian and non-Persian; education was classified as illiterate, less than diploma, and university. SES was calculated based on a latent variable measured by analysis of the participants’ assets using multiple correspondence analysis (MCA), and participants were categorized into quartiles of this variable (low, low-middle, middle-high, and high).

####  Disease Data 

 A total of 9264 individuals aged 40‒75 years participated in PCS. They were interviewed by a local physician and a trained nurse face to face. In this interview, the participants were asked, “Has your physician told you that you have the disease X and need treatment?”. If they answered “Yes”, they were categorized as having disease X.

 History of hypertension [HTN], diabetes mellitus (DM), heart disease, stroke, chronic obstructive pulmonary disease (COPD), sleep disorder, anxiety, depression, gastroesophageal reflux disease (GERD), irritable bowel syndrome (IBS), and functional constipation (FC) were obtained from the participants. Patients were asked about their symptoms to diagnose IBS, GERD, and FC, and ROME IV criteria were applied.

####  Drug Data

 Participants were asked to bring all their drugs with them during the interview. A trained nurse recorded the medications they had been taking for at least the past three months.

####  Drug Classification

 We used the first and second levels of the Anatomical Therapeutic Chemical (ATC) classification system to classify drugs. We merged drug classes with lower prevalence in the first level of ATC (e.g. Aot).

###  Test Methods

 Drugs used in each disease were listed based on UpToDate, and an expert panel consisting of two physicians and pharmacists reviewed the list to determine whether the drugs were included correctly or not. The use of drugs was defined as a binary variable for each disease in the analysis as a test. Each disease’s self-report was also considered a binary variable as a reference standard (Table 1). In this study, we utilized self-report data as a prevalent existing standard, despite its imperfections. This decision was informed by a substantial body of evidence that has examined the validity of self-report data in comparison to other standard references.^14-16^ However, if anyone believes that the reference standard should be the drug data, it can be achieved by referring to the description provided in Table 1. For those disorders for which self-report was not available in the PCS, only the estimated prevalence was derived from their drug use pattern (more details could be find in Table S1).

**Table 1:** Confusion Matrix and the Effects of Changing Standard Reference on Each Index

	**Drug Use**		
		**Positive Drug Use**	**Negative Drug Use**
Disease self-report	Positive self-report	TP, drug use and positive self-report (A)	FN, positive self-report but no drug use (B)
	Negative self-report	FP, positive drug use, negative self-report (C)	TN, neither positive self-report nor drug use (D)
	**Self-report as Reference Standard**		**Drug Data as Reference Standard**
$\frac{A}{A+B}$	Sensitivity		PPV
$\frac{D}{D+C}$	Specificity		NPV
$\frac{A}{A+C}$	PPV		Sensitivity
$\frac{D}{D+B}$	NPV		Specificity
$\frac{A}{A+B+C}$	Jaccard similarity index		Jaccard similarity index

FN, false negative; TP, true positive; TN, true negative; FP, false positive; PPV, positive predictive value; NPV, negative predictive value.

###  Statistical Methods

 Frequency (%) was used to describe the population and the pattern of drug use and diseases. We estimated the sensitivity, specificity, PPV, NPV, and 95% confidence interval (CI). We also used the Jaccard index to examine the similarities between disease self-report and drug use. The Jaccard index is the ratio of positive cases in both methods to the positive cases in either method.^17^ Therefore, when both test and standard reference methods report identically, the Jaccard index equals 100%, indicating the most similarity.^17^ The majority of the population is neither diseased nor takes any medication (TN). We chose the Jaccard similarity index to avoid the effect of the large TNs.


$$Jaccard\;Index=\frac{True\;postive}{True\;postive+False\;Negtive+False\;Postive}\times 100$$

## Results

 There were 9264 participants in PCS. More than half of them were women (53.85%). The age range of the participants was 40‒75 years. Almost half of the people were illiterate (Table 2).

**Table 2:** Demographic Characteristics and Comorbidities of Participants

**Variable**	**Frequency**
Age	
< 50	4209 45.40% (44.48‒46.51)
50-60	2825 30.47% (29.38‒31.26)
> 60	2236 24.12% (23.32‒25.07)
Gender	
Male	4277 46.15 % (45.13‒47.16)
Female	4991 53.85% (52.83‒54.86)
Ethnicity	
Non-Persian	4047 43.69% (42.68‒44.70)
Persian	5215 56.31% (55.29‒57.31)
Education	
Illiterate	4538 49.03% (48.01‒50.05)
Less than diploma	4436 47.93% (46.91‒48.94)
University	281 3.04% (2.70‒3.40)
Comorbidities	
None	1798 19.40% (18.60‒20.21)
One	2225 24% (23.14‒24.88)
Two or three	3278 35.36% (20.41‒22.08)

 Anxiety (29.63%), sleep disorders (19.37%), depression (19.38%), and HTN (16.34%) were the most common diseases in the population. The highest prevalences based on drug data were observed in heart disease (22%), GERD (18.9%), and HTN (16.1%). The highest pharmacotherapy rates were among heart disease (77.21%), HTN (69.6%), and diabetes (56.86%). The lowest pharmacotherapy rates were in IBS (2.44%), FC (2.5%), and depression (9.92%) (Table 3).

**Table 3:** Prevalence, Pharmacotherapy Rate and Performance Metrics of Self-report Versus Drug Use Data

**Disease**	**Drug ATC code**^a^	**Self-reported Prevalence** **(95% CI)**	**Drug Data Prevalence** **(95% CI)**	**Drug Use among Patients Who Reported Disease History (95% CI)**									**Jaccard index (95% CI)**	**PPV (95% CI)**	**NPV (95% CI)**	**Sensitivity (95% CI)**	**Specificity (95% CI)**
				**Male**			**Female**			**Total**							
				**<60**	**>60**	**Overall**	**<60**	**>60**	**Overall**	**<60**	**>60**	**Overall**					
HTN	C07 C08 C09 C03 C02	16.34 (15.6 ‒17.1)	16.1 (15.4‒16.9)	80.8 (75.3‒86.5)	63.4 (57.3‒69.6)	71.1 (66.9‒75.5)	66.6 (62.8‒70.6)	71.5 (67.7‒75.4)	68.9 (66.3‒71.8)	70.1 (67‒73.5)	69.0 (65.8‒72.3)	69.6 (67.3‒72)	53(50.8 ‒55.2)	70.25 (67.9‒72.6)	94.08 (93.6‒94.6)	69.6 (67.3 ‒71.9)	94.25 (93.7‒94.8)
DM	A10	9.44 (8.9‒10.1)	5.7‒6.2)	71.43 (63.8‒79.2)	44.29 (36.1‒52.6)	57.51 (51.7‒63.4)	56.73 (51.6‒62)	56.35 (50.3‒62.5)	56.57 (52.7‒60.6)	60.79 (56.5‒65.2)	52.04 (47.1‒57)	56.86 (53.6‒60.2)	54(50.8 ‒57.2)	93.25 (91.1‒95.4)	95.69 (95.3‒96.1)	56.86 (53.6 ‒60.1)	99.57 (99.4‒99.7)
Heart disease	C07 C10 C01 C08 C09 C03 B01	10.38 (9.8‒11.1)	22 (21.2‒22.9)	84.69 (79.7‒89.8)	65.38 (59‒71.9)	74.75 (70.6‒79)	77.41 (72.5‒82.4)	80.49 (76‒85.1)	78.99 (75.7‒82.4)	80.47 (76.9‒84.1)	74.14 (70.3‒78)	77.21 (74.6‒79.9)	32(30.1 ‒33.9)	36.28 (34.2‒38.4)	96.97 (96.6‒97.4)	77.21 (74.6 ‒79.9)	84.32 (83.5‒85.1)
Stroke	C10 B01	1.79 (1.6‒2.1)	10.8 (10.2‒11.5)	29.6(-2 ‒61.3)	39.6(17.5 ‒61.6)	36(17.8 ‒54.2)	35.7(11.4 ‒60)	42.9 (21.6‒64.1)	39.6 (23.5 ‒55.6)	33.3(14 ‒52.6)	41.2(25.9 ‒56.5)	38(25.9‒50)	9.5(8.2 ‒10.8)	68.7 (63.1‒74.4)	82.1 (81.3‒82.8)	9.9(8.5 ‒11.3)	98.9 (98.7‒99.2)
GERD (Rome IV)^c^	A02 Aot^b^	8.65 (8.1‒9.3)	18.9 (18.1‒19.7)	51.54 (43‒60.2)	43.1 (30.4‒55.9)	48.94 (41.8‒56.1)	37.67 (33.2‒42.3)	48.86 (41.5‒56.3)	40.88 (37‒44.8)	40.85 (36.9 ‒44.9)	47.44 (41.1‒53.9)	42.77 (39.4‒46.2)	15(13.5 ‒16.5)	19.56 (17.7‒21.4)	93.89 (93.3‒94.4)	42.77 (39.3 ‒46.2)	83.34 (82.5‒84.1)
IBS (Rome IV)^c^	Aot^b^ A06 A07	11.64 (11‒12.3)	1.4 (1.1‒1.6)	6.92 (2.6‒11.3)	0.82 (0‒2)	2.94 (1.3‒4.7)	2.74 (1.3‒4.3)	1.18 (0‒2.6)	2.16 (1.1‒3.3)	3.7 (2.2‒5.3)	1 (0.2‒1.9)	2.44 (1.6‒3.4)	2 (1.2 ‒2.8)	20 (13.1‒26.9)	88.61 (88‒89.3)	2.44(1.5 ‒3.4)	98.73 (98.5‒99)
FC (Rome IV)^c^	A06	8.1 (7.5‒8.6)	0.4 (0.3‒0.6)	3.1(0‒22.6)	2.1(0‒16.3)	2.5(0‒14)	2.2(0‒14.1)	3.1(0‒16.9)	2.6 (0‒11.6)	2.5(0‒ 12.6)	2.6(0‒12.5)	2.5(0‒9.6)	2.4(1.4 ‒3.5)	44.2 (29.3‒59)	92 (91.5‒92.6)	2.5(1.4 ‒3.6)	99.7 (99.6‒99.8)
COPD	R03	3.21 (2.9‒3.6)	1.3 (1.1‒1.5)	52.63 (39.7‒65.6)	62.07 (44.5‒79.8)	55.81 (45.4 ‒66.4)	27.63 (20.6‒34.8)	36.67 (24.5‒48.9)	30.19 (24.1‒36.4)	34.45 (28.1‒ 40.9)	44.94 (34.7‒55.3)	37.58 (32.1‒43.1)	30(24.9 ‒35.1)	91.06 (86‒96.1)	97.97 (97.7‒98.3)	37.58 (32.1 ‒43.1)	99.88 (99.8‒100)
Sleep disorder	N05B N05A N06A	19.37 (18.6‒20.2)	5.6 (5.1‒6)	25.8(14.3 ‒37.3)	5.7(-3.7 ‒15.3)	12.8(5.4 ‒20.2)	16.9(10.4 ‒23.5)	11.7 (2.7‒20.7)	15 (9.7‒20.4)	18.9(13.2 ‒24.6)	8.8(2.3 ‒15.4)	14.3(9.9 ‒18.6)	12.4(11 ‒13.9)	49.2 (44.9‒53.5)	82.4 (81.6‒83.2)	14.3(12.6 ‒15.9)	96.5 (96‒96.9)
Anxiety	N05B N06A N05C C07A A05	29.63 (28.7‒30.6)	8.1 (7.6‒8.7)	26.5(17.3 ‒35.6)	6(-2.9‒15)	14.9(8.4 ‒21.4)	20.6(15.8 ‒25.5)	14.9 (7.6‒22.2)	18.8(14.8 ‒22.9)	21.8(17.5 ‒26.1)	11.2(5.4 ‒16.9)	17.7(14.2 ‒21.1)	14.6 (13.4 ‒15.8)	64.1 (60.7‒67.5)	70.9 (70‒71.9)	15.9(14.6 ‒17.2)	95.8 (95.3‒96.3)
Depression	N05A N06A	19.38 (18.6‒20.2)	2.7 (2.4‒3.1)	18.14 (13.3‒23.1)	1.66 (0.3‒3.2)	8.91 (6.6‒11.4)	12.03 (9.9‒14.3)	7.06 (4.7‒9.5)	10.35 (8.7‒12.1)	13.39 (11.4‒15.5)	4.81 (3.3‒6.4)	9.92 (8.6‒11.4)	9 (7.7‒10.3)	68.73 (63.1‒74.4)	82.06 (81.3‒82.9)	9.92(8.5 ‒11.3)	98.92 (98.7‒99.2)

^a^ Supplementary file 1 contains the names of drugs used within each ATC group. ^b^ Other subclasses of A group witsh lower use ^c^ In these diseases, patients were asked about their symptoms and were diagnosed based on ROME IV criteria.

 The highest similarity between self-reported diseases and drug data was observed in HTN (53%) and DM (54%). In patients with IBS, FC, and depression, the similarity index was 2%, 2.4%, and 9%, respectively. Drug use in patients with DM (93.2%), COPD (91%), and HTN (72%) indicated the highest PPV. PPV was 19.5%, 20%, and 44.2% in patients with GERD, IBS, and FC. Reported NPVs were all above 70%. The sensitivity of drug data in patients identified with heart disease, HTN, and DM were 77.2%, 69.6%, and 56.86%, respectively. IBS, FC, depression, and sleep disorders sensitivity was reported at 2.44%, 2.5%, 9.92%, and 14.3% (Table 3).

## Discussion

 In this study, we aimed to investigate the similarity of self-report data of diseases and drug data. We found that in most cases, drug data show low similarity to the self-report data. To estimate the prevalence of diseases in our setting, however, drug data does not seem to be a suitable tool to use for patients with IBS, GERD, FC, Stroke, COPD, sleep disorders, diabetes anxiety, heart disease, and depression. The prevalence of diseases based on drug data was either equally, over- or under-reported compared to the self-report data. So, our hypothesis that drug data are suitable for estimating the prevalence of diseases in less-developed settings seems to be rejected.

 DM was under-reported by drug data, possibly because of the low medication adherence rate, which leads to less drug use in diseased individuals.^18^ Low disease awareness, perception of illness, and health-seeking behavior were also reported as factors contributing to the low rate of self-report of diseases.^19,20^ Moreover, a study based on pharmacy claim data in Iran reported that the DM prevalence rate was 6.4%, similar to our results^4^. Drug data also underreported COPD. Complex treatment regimens contributed to low medication adherence in COPD patients.^21,22^ Previous studies also indicated moderate health literacy in Iranian COPD patients,^23^ and health literacy correlated with medication adherence.^24^ In both cases of DM and COPD, PPV was above 90%, demonstrating that positive drug use data is a good predictor of self-report. A similar study indicated that the prevalence of COPD drug use was 5.2%,^4^ which may be due to population variation. The mentioned study used data from both urban and rural areas.

 Drug data under-report IBS and FC prevalence. In both IBS and FC similarity index, sensitivity and PPV are low. Both these diseases were diagnosed based on ROME IV criteria through the interview; therefore, many patients were not diagnosed before the session and were not treated. Treatment of these diseases consists of lifestyle and dietary modification, physical activity, and pharmacotherapy.^25^ Accordingly, not all patients receive medication,^26,27^ leading to high false negative (FN). Over-the-counter (OTC) access to these drugs in patients with other differential diagnoses is probably the cause of drug use in ROME IV-negative individuals, contributing to high false positive (FP).

 Drug data under-report the prevalence of sleep disorders, anxiety, and depression. Similarity index, sensitivity, and PPV are low, which implies high FN and FP. The high number of patients with these diseases who do not use medication may be caused by undertreatment,^28,29^ low medication adherence,^30,31^ and other non-pharmacological treatments such as cognitive behavioral therapy. Drugs used in treating these diseases overlap with each other and other mental disorders. Social stigma for mental disorders may also affect self-report of these diseases.^32^

 The prevalence of self-reported estimates and the drug data are similar in HTN. Sensitivity, PPV, and similarity index are low, remarking that the individuals identified by each are different. Drugs used in HTN are commonly used in other diseases like heart disease and stroke. Also, many hypertensive patients are undiagnosed, insufficiently treated, or have low compliance.^19^ Therefore, the mentioned indices are low.

 Drug data over-report heart disease, stroke, and GERD. Drugs used to treat heart disease, stroke, and HTN overlap with each other. In another study, these three diseases were reported as a pooled group of cardiovascular diseases.^4^ It seems that drug data cannot provide the prevalence of the overlapped diseases separately. GERD symptoms are common in the population, overlap with other differential diagnoses, and its drugs are available OTC. Patients with minimal symptoms self-medicate,^33^ while ROME IV criteria are not met. DM and COPD are the two diseases that have no overlap with other diseases assessed here in terms of medical therapy.

 The pharmacotherapy rate among COPD patients varies significantly between genders; the male pharmacotherapy rate was higher than the female, probably due to the higher prevalence, severity, and diagnosis of COPD in men, as reported.^34^ Pharmacotherapy decreased with aging in men except in COPD and stroke. In women, aging increased the pharmacotherapy rate in most diseases except for IBS and mental disorders. So, these results show that the pharmacotherapy rate varies across demographic groups and diseases.

 Controversy exists concerning self-report validity in the literature.^7,8^ Some studies showed that self-reporting had a substantial agreement with health records or under-reporting of the prevalence of diseases. In one study, Smith et al compared self-reporting of diseases to health records and found that it is better to use self-report data to rule out the diseases and use more objective data for prevalence studies.^10^ Self-report data is thought to be affected by recall bias, desirability bias,^1^ cognitive deficit, and language barrier. Drug data, a more objective data source, seems less influenced by these shortcomings. It should be considered that drug data itself can be affected by many factors, including adherence to medications, knowledge, attitude, and practice of physicians, access to health services, and pharmacotherapy rate of the disease. The treatment strategy of the diseases influences the disease pharmacotherapy rate. For example, in diseases where non-pharmacological interventions such as lifestyle and dietary modifications and cognitive behavioral therapy are used commonly, drug data cannot be used to estimate the prevalence of those diseases. Also, different health-seeking behaviors across diverse demographic groups lead to different pharmacotherapy rates; this may lead to overestimation or underestimation in different population groups, which can affect the representativeness and generalizability of the drug data results. It should be kept in mind that using the same drug for different diseases or off-label use of drugs can also interfere with prevalence estimation. This could lead to the overestimation of some diseases. Thus, combining drug data with self-report data can be a suitable alternative to using either alone.

 We only compared self-report data with drug data. Using other data sources, such as health records, provides us with a more accurate understanding of drug data’s ability to estimate disease prevalence. So, we acknowledge that utilizing health records as a means to compare drug data could potentially offer advantages. However, it is important to note that the unavailability of such data is a shared limitation in our setting and other regions of Iran. We used large groups of drugs (1^st^ and 2^nd^ level of ATC) in this study. It is assumed that using large groups of drugs can lead to overestimation. However, our results indicated an underestimation in prevalence and low sensitivity of drug data in most diseases. So, if smaller groups of drugs were applied, the gap between drug data and self-report data would have become even wider. We also considered self-report data as the reference standard to compare these two data sources. Although self-report has several biases, we use the similarity index, which is not affected by changing the reference standard. Other indices we use in our study could also be converted to each other if the reference standard changes (Table 1).

## Conclusion

 In conclusion, self-reports of diseases and the drug data show a different picture of most diseases’ prevalence in our setting. It seems that drug data alone cannot estimate the prevalence of diseases in settings similar to our study. We recommend using drug data in combination with self-report data for epidemiological investigation in the less-developed setting.

## Supplementary Files


Supplementary file 1 contains Table S1.
