# Supplementary material for: How Similar Are Drug Data and Disease Self-report? Estimating the Prevalence of Chronic Diseases in Less Developed Settings
Source: Arch Iran Med. 2024 Jul 1;27(7):364–70. doi: 10.34172/aim.27553 (PMC11316190; doi:10.34172/aim.27553)
Supplement: Supplementary file 1 — contains Table S1. [file aim-27-364-s001.pdf]

Arch Iran Med. July 2024;27(7):364-S1

10.34172/aim.27553

Supplementary

For those disorders for which self-report was not available in the PCS, only the estimated prevalence was derived from their drug use pattern. In these diseases, the prevalence was extracted based on the best-published evidence (meta-analysis, if available) from Iran with a similar conduction date compared to the cohort. If prevalence intervals intersect each other, we consider them similar.

Supplement Table 1. Prevalence of Diseases base on drug use pattern and published literature

Disease	Drugs Name	%	Prevalence Based on Published Literature %$	Reference
**Hypothyroidism**	Levothyroxine	1.7 (1.5–2)	3.7–12.8%	1–4
**Hyperthyroidism**	Thiamazole	1 (0.8–1.3)	0.23–4.63%	5
**Parkinson**	Levodopa<br>Trihexyphenidyl<br>Amantadine<br>Pramipexole<br>Selegiline<br>Benserazide<br>Bromocriptine<br>Rivastigmine	0.19 (0.1–0.3)	0.05–0.26%	6
**Hepatitis B**	Tenofovir<br>Adefovir<br>Interferon alfa<br>Lamivudine	0.1(0.04–0.18)	2.2–9%	7–9
**Hyperlipidemia**	Atorvastatin<br>Lovastatin<br>Simvastatin<br>Gemfibrozil<br>fenofibrate<br>Clofibrate	7.5(7–8)	35.5–46%	10,11
**Osteoporosis**	Alendronic acid<br>Calcium and Vitamin D	2 (1.7–2.3)	13–20%	12
**Osteoarthritis**	Glucosamine<br>Preparation<br>Piascledine	0.2 (0.1–0.3)	14–53%	13–15
**Migraine**	Sumatriptan<br>Rizatriptan<br>Ergotamine	0.4 (0.3–0.6)	7.14–18.11%	16,17
**Iron Deficiency**	Iron supplement	13.5 (12.8–14.2) *	13.6–27.7%	18,19

*Prevalence in female gender 24.12(22.94-25.34%)

The prevalence of hyperthyroidism was estimated to be 1% (0.8–1.3%), Parkinson's disease 0.19% (0.1–0.3%), iron deficiency 13.5% (12.8–14.2%), and hyperlipidemia 7.5%(7–8%) based on drugs used in the population (Supplement Table). Self-reports of the diseases listed in Supplement Table 1 are not included in PCS data.

References

1. Aminorroaya A, Janghorbani M, Amini M, Hovsepian S, Tabatabaei A, Fallah Z. The prevalence of thyroid dysfunction in an iodine-sufficient area in Iran. Archives of Iranian medicine. 2009;12(3):262-70. Epub 2009/04/30. PubMed PMID: 19400604.

2. Delshad H, Mehran L, Tohidi M, Assadi M, Azizi F. The incidence of thyroid function abnormalities and natural course of subclinical thyroid disorders, Tehran, I.R. Iran. Journal of endocrinological investigation. 2012;35(5):516-21. Epub 2011/10/06. doi: 10.3275/7968. PubMed PMID: 21971483.

3. Mahdavi M, Amouzegar A, Mehran L, Madreseh E, Tohidi M, Azizi F. Investigating the prevalence of primary thyroid dysfunction in obese and overweight individuals: Tehran thyroid study. BMC Endocrine Disorders. 2021;21(1):1-12.

4. Niafar M, Aliasgharzadeh A, Bahrami A, Najafipur F, editors. Prevalence of thyroid dysfunction in the elderly women of Iran. Endocrine Abstracts; 2009: Bioscientifica.
5. Sajjadi-Jazi SM, Sharifi F, Varmaghani M, Meybodi HA, Farzadfar F, Larijani B. Epidemiology of hyperthyroidism in Iran: a systematic review and meta-analysis. Journal of Diabetes & Metabolic Disorders. 2018;17(2):345-55. doi: 10.1007/s40200-018-0367-1.
6. Hosseinzadeh A, Baneshi MR, Sedighi B, Kermanchi J, Haghdoost AA. Estimation of Parkinson's Disease Prevalence and Its Geographical Variation in Iran. Journal of Mazandaran University of Medical Sciences. 2021;31(200):113-24.
7. Kalvandi G, Abangah G, Veisani Y, Nourmohammadi H, Golitaleb M, Tavan H. The frequency, related cause of disease, and treatment of hepatitis B virus infection: A systematic review and meta-analysis in Iran. Journal of Research in Medical Sciences. 2022;27(1):15-. doi: 10.4103/jrms.JRMS_67_19.
8. Mohammadi Z, Keshtkar A, Eghtesad S, Jeddian A, Pourfatholah AA, Maghsudlu M, et al. Epidemiological Profile of Hepatitis B Virus Infection in Iran in the Past 25 years; A Systematic Review and Meta-analysis of General Population Studies. Middle East journal of digestive diseases. 2016;8(1):5-18. Epub 2016/03/05. doi: 10.15171/mejdd.2016.01. PubMed PMID: 26933476; PubMed Central PMCID: PMCPMC4773083.
9. Salehi-Vaziri M, Sadeghi F, Almasi Hashiani A, Gholami Fesharaki M, Alavian SM. Hepatitis B Virus Infection in the General Population of Iran: An Updated Systematic Review and Meta-Analysis. 2016;16(4):e35577. Epub 2016-04-02. doi: 10.5812/hepatmon.35577.
10. Tabatabaei-Malazy O, Qorbani M, Samavat T, Sharifi F, Larijani B, Fakhrzadeh H. Prevalence of dyslipidemia in iran: a systematic review and meta-analysis study. Int J Prev Med. 2014;5(4):373-93. Epub 2014/05/16. PubMed PMID: 24829725; PubMed Central PMCID: PMCPMC4018586.
11. Akbartabar Toori MP, Kiani FM, Sayehmiri FP, Sayehmiri KP, Mohsenzadeh YM, Ostovar RP, et al. Prevalence of Hypercholesterolemia, High LDL, and Low HDL in Iran: A Systematic Review and Meta-Analysis. Iranian journal of medical sciences. 2018;43(5):449-65. Epub 2018/09/15. PubMed PMID: 30214097; PubMed Central PMCID: PMCPMC6123550.
12. Irani AD, Poorolajal J, Khalilian A, Esmailnasab N, Cheraghi Z. Prevalence of osteoporosis in Iran: A meta-analysis. Journal of research in medical sciences : the official journal of Isfahan University of Medical Sciences. 2013;18(9):759-66. Epub 2014/01/02. PubMed PMID: 24381618; PubMed Central PMCID: PMCPMC3872583.
13. Kolahi S, Khabbazi A, Malek Mahdavi A, Ghasembaglou A, Ghasembaglou A, Aminisani N, et al. Prevalence of musculoskeletal disorders in Azar cohort population in Northwest of Iran. Rheumatology international. 2017;37(4):495-502. Epub 2017/02/09. doi: 10.1007/s00296-017-3661-1. PubMed PMID: 28168545.
14. Davatchi F, Jamshidi AR, Banihashemi AT, Gholami J, Forouzanfar MH, Akhlaghi M, et al. WHO-ILAR COPCORD Study (Stage 1, Urban Study) in Iran. The Journal of rheumatology. 2008;35(7):1384. Epub 2008/05/09. PubMed PMID: 18464299.
15. Tehrani-Banihashemi A, Davatchi F, Jamshidi AR, Faezi T, Paragomi P, Barghamdi M. Prevalence of osteoarthritis in rural areas of Iran: a WHO-ILAR COPCORD study. International journal of rheumatic diseases. 2014;17(4):384-8. Epub 2014/03/13. doi: 10.1111/1756-185x.12312. PubMed PMID: 24618176.
16. Farhadi Z, Alidoost S, Behzadifar M, Mohammadibakhsh R, Khodadadi N, Sepehrian R, et al. The Prevalence of Migraine in Iran: A Systematic Review and Meta-Analysis. Iranian Red Crescent medical journal. 2016;18(10):e40061. Epub 2017/02/12. doi: 10.5812/ircmj.40061. PubMed PMID: 28184330; PubMed Central PMCID: PMCPMC5292015.
17. Sadeghi O, Nasiri M, Saiedi SG. The Prevalence of Migraine in Different Parts of Iran: Review of the Current Evidence. 2015;4(3):e27678. Epub 2015-07-02. doi: 10.5812/jjcdc.4(3)2015.27678.
18. Esmat B, Mohammad R, Behnam S, Shahrzad M, Soodabeh T, Minoo A, et al. Prevalence of Iron Deficiency Anemia among Iranian Pregnant Women; a Systematic Review and Meta-analysis. Journal of reproduction & infertility. 2010;11(1):17-24. Epub 2010/04/01. PubMed PMID: 23926476; PubMed Central PMCID: PMCPMC3719272.
19. Akbari M, Moosazadeh M, Tabrizi R, Khatibi SR, Khodadost M, Heydari ST, et al. Estimation of iron deficiency anemia in Iranian children and adolescents: a systematic review and meta-analysis. Hematology (Amsterdam, Netherlands). 2017;22(4):231-9. Epub 2016/10/16. doi: 10.1080/10245332.2016.1240933. PubMed PMID: 27741938.
